# Supplementary figures and images for: Natural variation in temperature-modulated immunity uncovers transcription factor bHLH059 as a thermoresponsive regulator in Arabidopsis thaliana
Source: PLoS Genet. 2021 Jan 25;17(1):e1009290. doi: 10.1371/journal.pgen.1009290 (PMC7861541; doi:10.1371/journal.pgen.1009290)

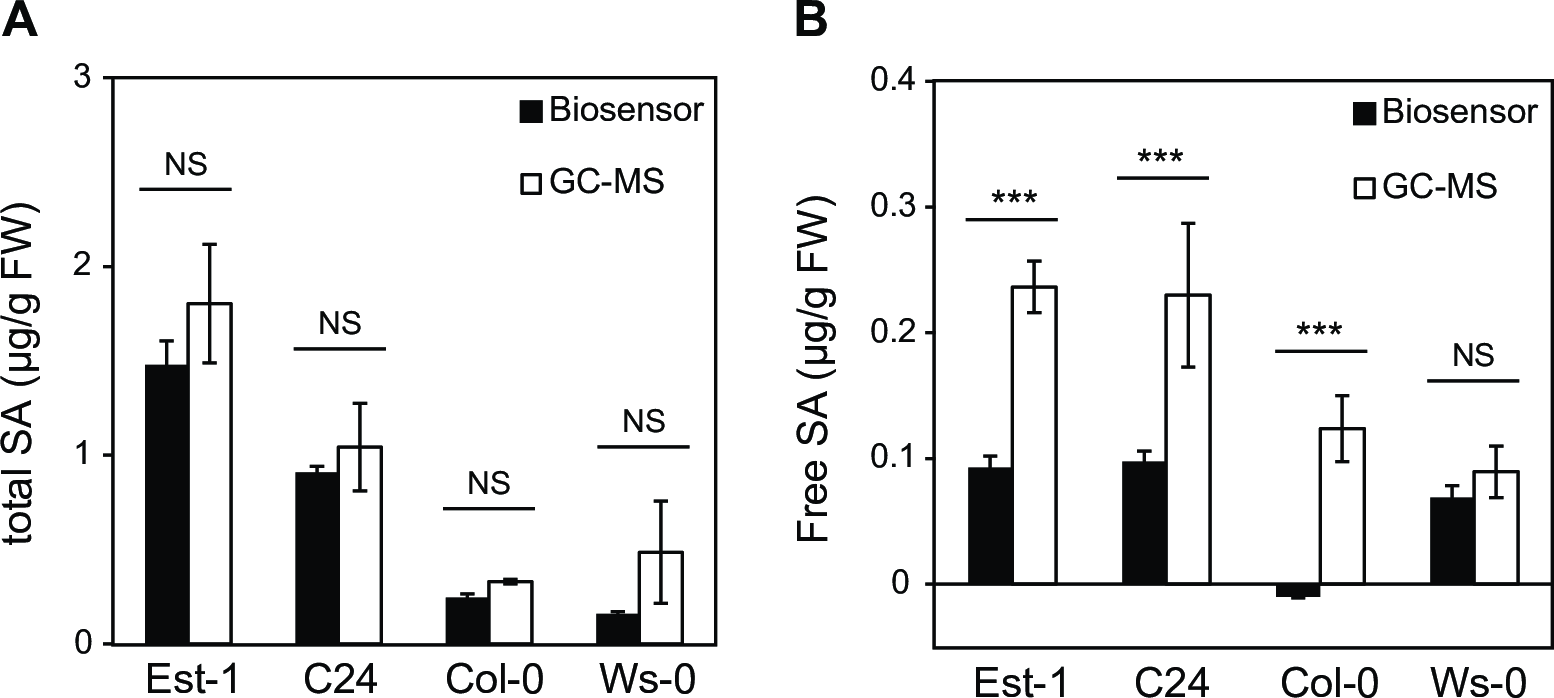

Supplement: S1 Fig — A) Total SA in four A. thaliana accessions with contrasting SA contents grown at 22°C (n = 5 replicates from one experiment). Significant differences between methods after Student t-test with p-value<0.05 are indicated with stars on plot. NS = not significant. B) Free SA in four A. thaliana accessions with contrasting SA contents grown at 22°C (n = 5 replicates from one experiment). Significant differences between methods after Student t-test with p-value<0.05 are indicated on plot. NS = not significant. (TIF) [file pgen.1009290.s005.tif]

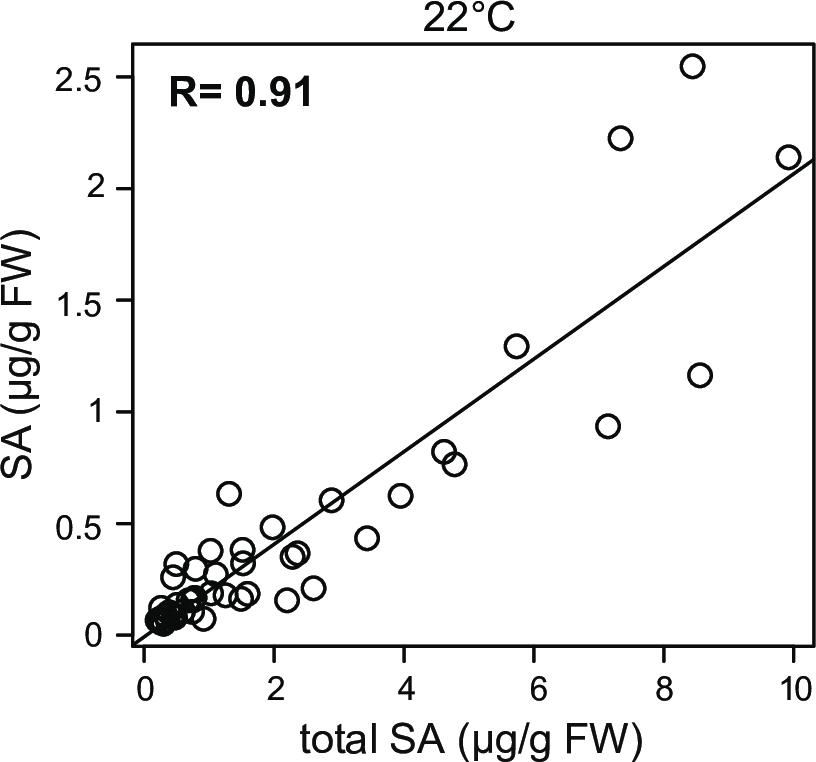

Supplement: S2 Fig — Plants were 5-week-old when sampled and grown at 22°C. R = Pearson’s correlation index (t = 14.365, df = 43, p-value < 2.2e-16). (TIF) [file pgen.1009290.s006.tif]

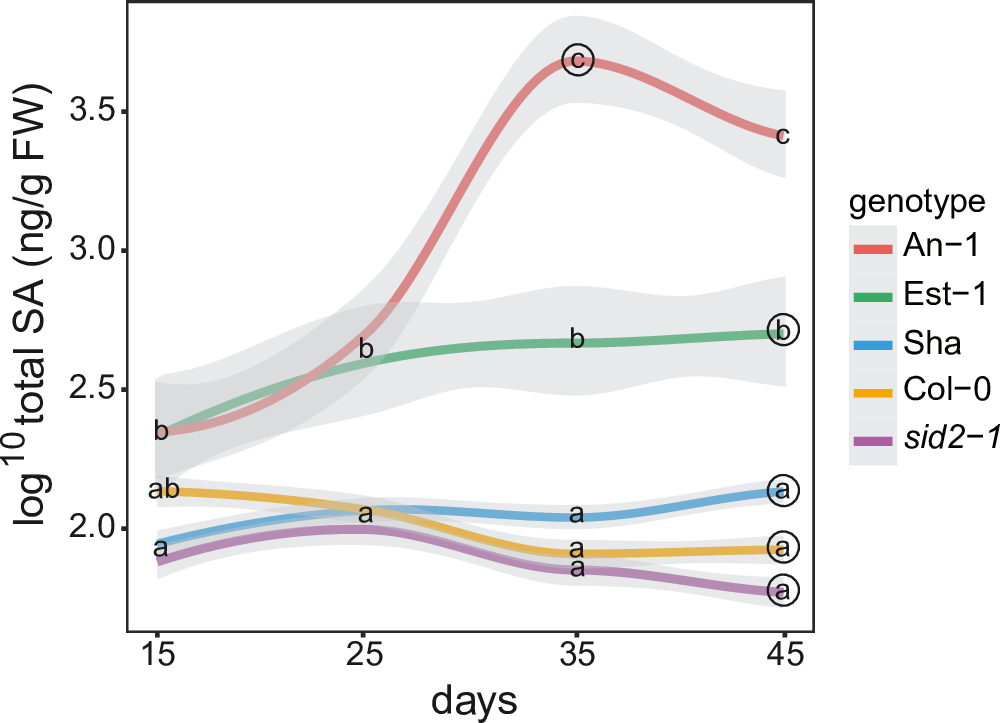

Supplement: S3 Fig — Letters indicate significant differences after Tukey’s multiple testing correction in one-way ANOVA. Circles indicate time point at which 100% plants were flowering. Grey shadows indicate standard error. (TIF) [file pgen.1009290.s007.tif]

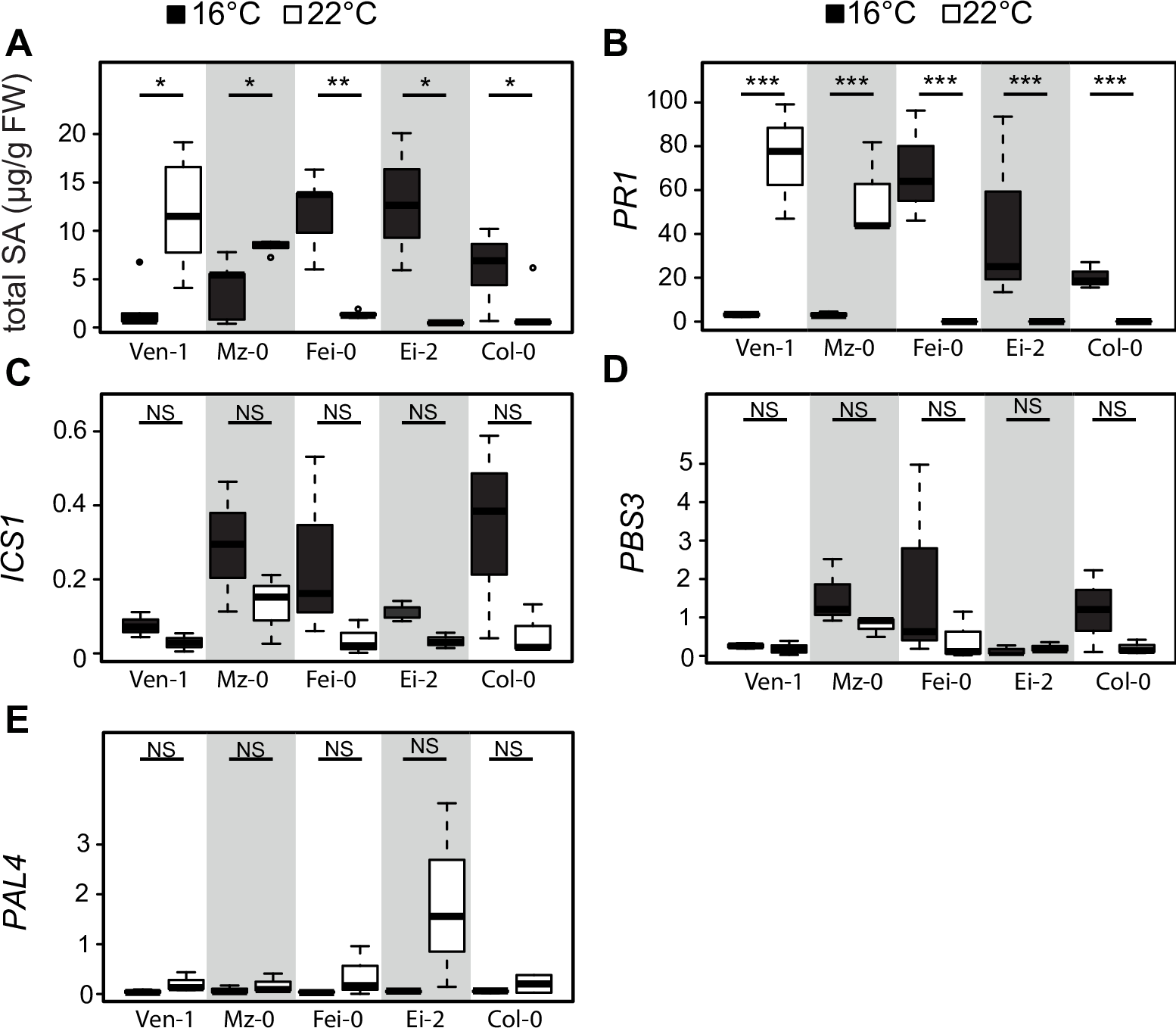

Supplement: S4 Fig — A) Total SA in Ven-1, Mz-0, Fei-0, Ei-2 and Col-0 measured by GC-MS. Statistical differences according to Kruskal–Wallis rank sum test with p-value<0.05 are indicated with stars. N = 5 independent biological replicates except for Ei-2 where n = 3 B) PR1, C) ICS1, D) PBS3, and E) PAL4 expression in Ven-1, Mz-0, Fei-0, Ei-2 and Col-0. Statistical differences according to Student t-test or Kruskal–Wallis rank sum test with p-value<0.05 between temperatures within each genotype are indicated with stars or NS = non significant on the graphic. N = 3 independent biological replicates. (TIF) [file pgen.1009290.s008.tif]

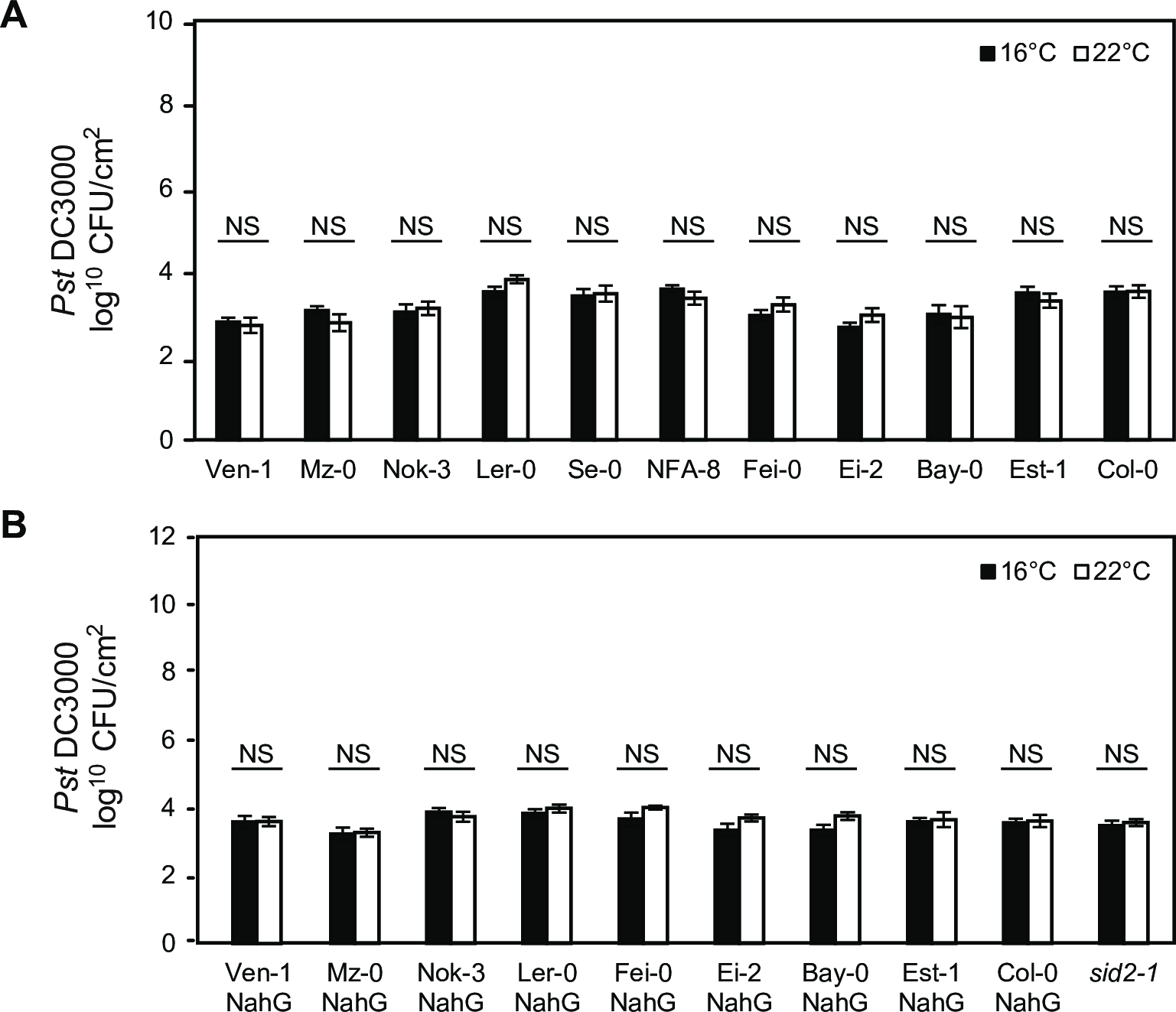

Supplement: S5 Fig — A) Bacteria-inoculated 5-week-old plants of 11 A. thaliana accessions, as indicated, grown at 16°C or 22°C (n = 9, three biological replicates). Significant differences between temperatures after Student t-test with p-values < 0.05 are indicated on plot with a star. NS = not significant. Error bars represent standard error. B) Bacteria-inoculated 5-week-old plants of 10 SA-deficient A. thaliana accessions grown at 16°C or 22°C (n = 9 from 3 biological replicates except for Ven-1 where n = 6). Significant differences between temperatures after Student t-test with p-values < 0.05 are indicated on plot with a star. NS = not significant. Error bars represent standard error. (TIF) [file pgen.1009290.s009.tif]

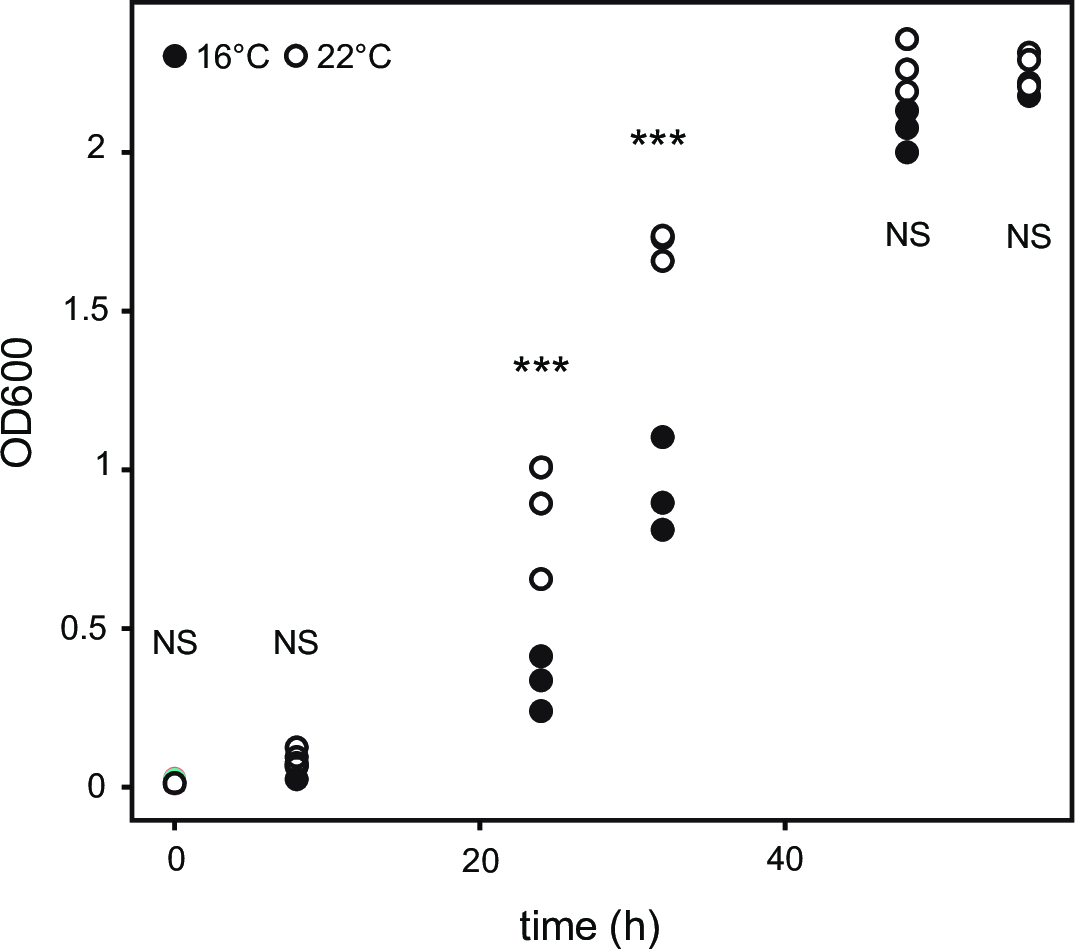

Supplement: S6 Fig — Bacteria were measured by optical density (OD600) increase over 56 h in M9 minimal salt medium with sorbitol at 16°C (black) and 22°C (white) (n = 3 from three biological replicates). Significant differences after Student t-test with p-value<0.05 are represented with stars. NS = not significant. (TIF) [file pgen.1009290.s010.tif]

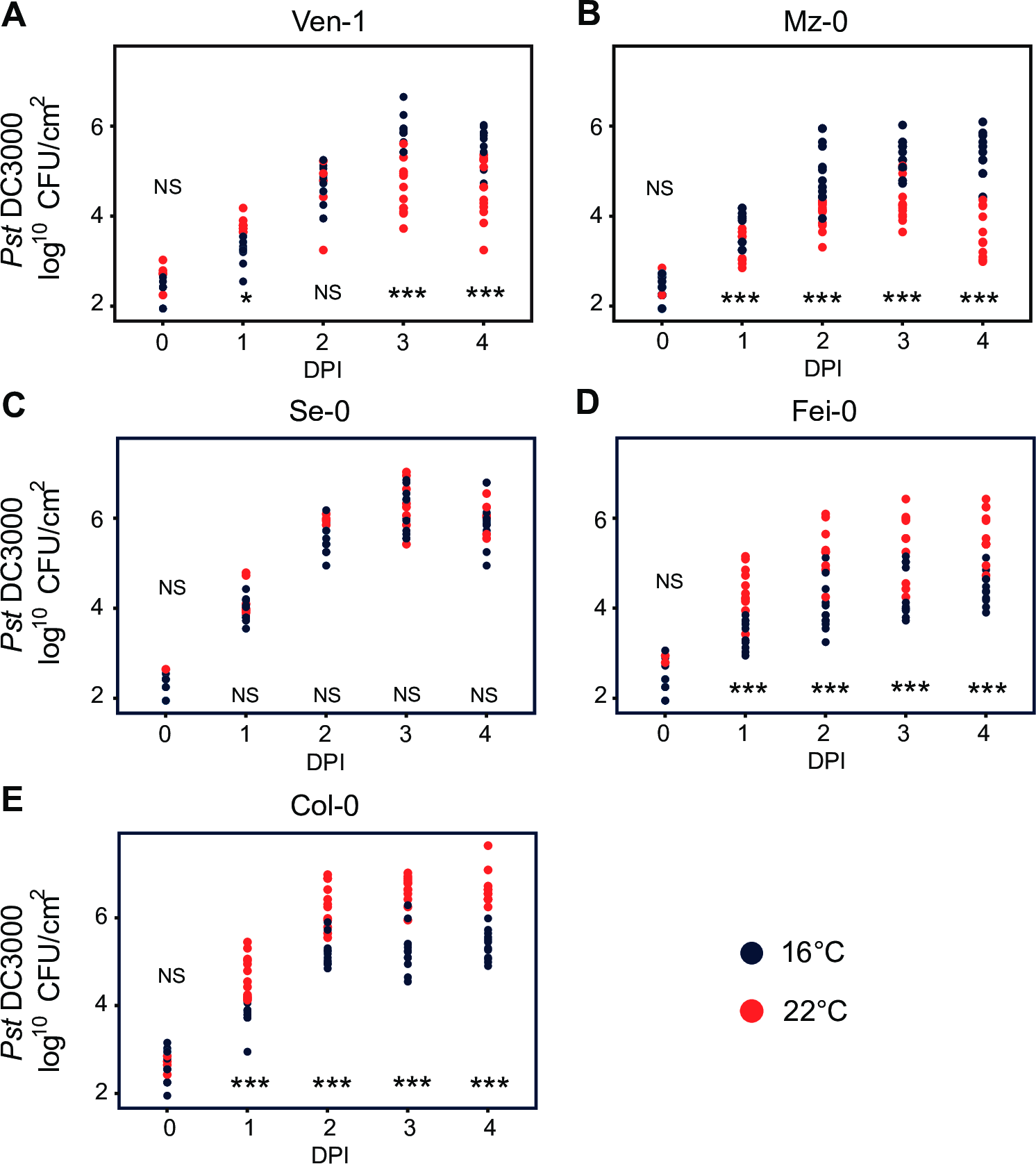

Supplement: S7 Fig — A) Ven-1 B) Mz-0 C) Se-0 D) Fei-0 E) Col-0. N = 12 for each time point by temperature and by genotype including four replicates for each of three independent biological replicates. Significant differences between temperature regimes at each time point with p-value<0.05 are indicate with stars on the graphic. NS = not significant. (TIF) [file pgen.1009290.s011.tif]

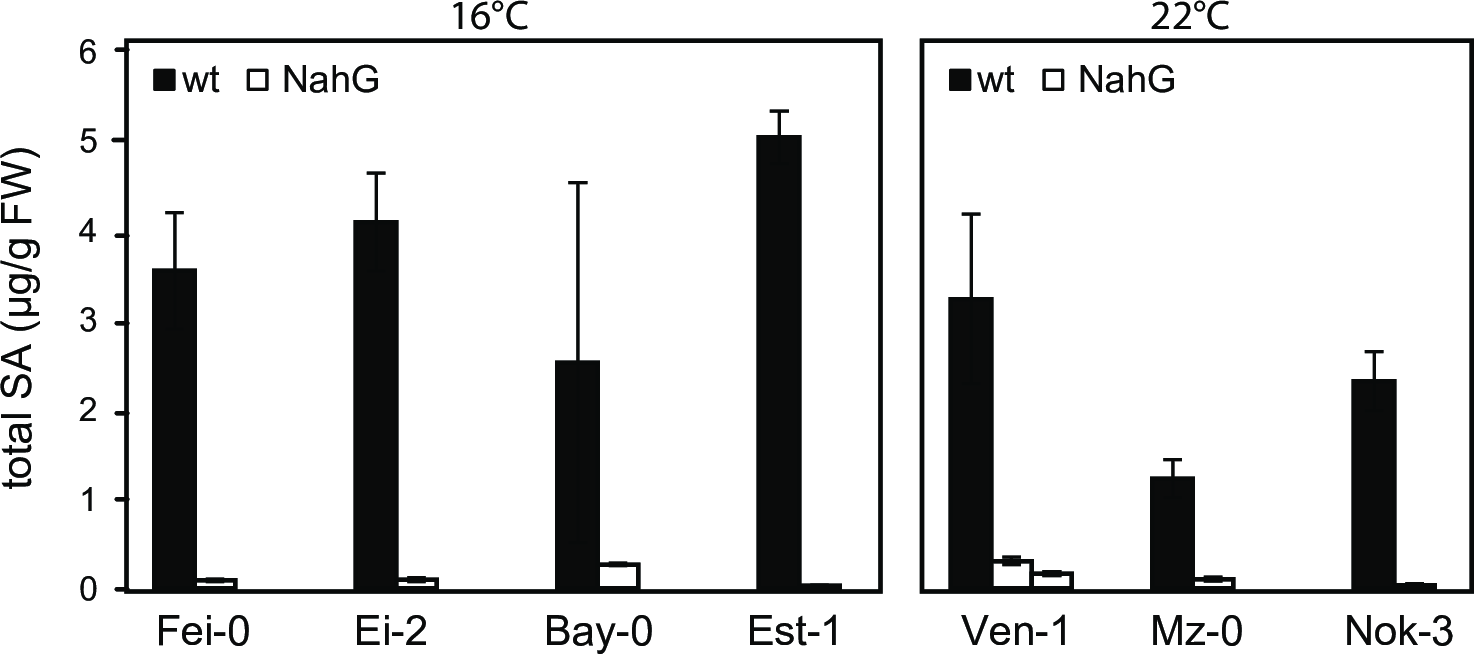

Supplement: S8 Fig — Transgenic lines are homozygous except for Ven-1(due to a long generation time) for which two heterozygous lines were tested.Lines were phenotyped in the environment in which the parental line displayed highest SA accumulation to ensure full SA depletion (n = 3 from 3 biological replicates). Error bars represent standard error. (TIF) [file pgen.1009290.s012.tif]

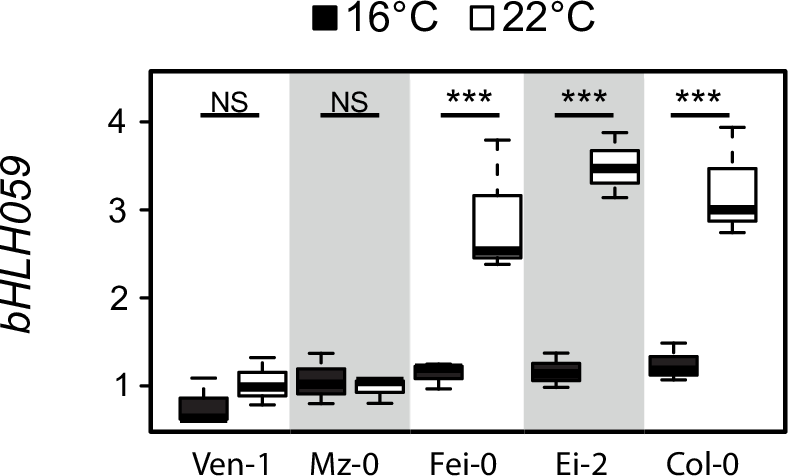

Supplement: S9 Fig — Differential expression in response to temperature according to student t-test with p-value<0.05 is indicated with stars on graphic. NS = not significant. (TIF) [file pgen.1009290.s013.tif]

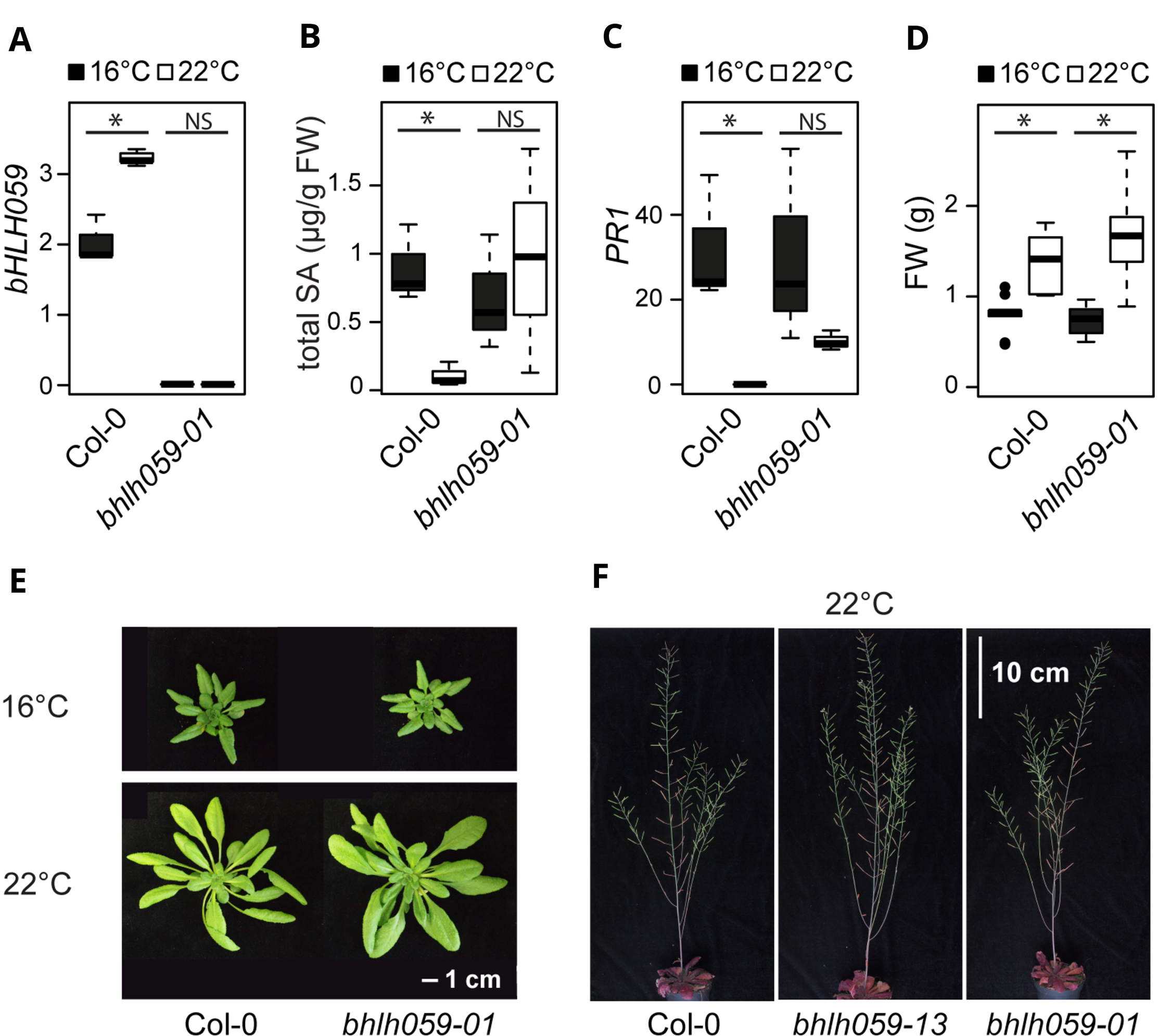

Supplement: S10 Fig — Data are represented as boxplots. Significant differences after student t-test with p-value<0.05 are represented with a star. NS = not significant. A) bHLH059 expression relative to SAND reference gene in mature leaves (n = 3 from biological replicates). B) Total SA contents in mature leaves (n = 3 from biological replicates). C) PR1 expression levels relative to SAND in mature leaves (n = 3 from biological replicates). D) Above-ground fresh weight (n = 3 from 3 biological replicates). e) Visual phenotypes of lines at 16°C and 22°C. F) Inflorescence with mature siliques of Col-0 and bHLH059 mutant lines. (TIF) [file pgen.1009290.s014.tif]
