# Supplementary material for: Natural variation in temperature-modulated immunity uncovers transcription factor bHLH059 as a thermoresponsive regulator in Arabidopsis thaliana
Source: PLoS Genet. 2021 Jan 25;17(1):e1009290. doi: 10.1371/journal.pgen.1009290 (PMC7861541; doi:10.1371/journal.pgen.1009290)
Supplement: Attachment — Submitted filename: Response to ReviewersBruessow.docx [file pgen.1009290.s015.docx]

Dear Dr. Mauricio,
Thanks to you and the reviewers for the helpful comments on our manuscript and allowing us more time to address them experimentally. We submit here a revised manuscript with major changes. All changes to manuscript are marked in yellow. In summary:

1. To address the possible role of stomatal resistance dynamics in altering bacterial growth in leaves of different accessions in response to temperature, we performed a bacterial apoplastic infiltration experiment with additional time points in five representative differential *A. thaliana* accessions at 16^o^C and 22^o^C. Data are shown in **new Fig S7**. Based on this and our existing data (Fig. 3) we argue that apoplastic (post-stomatal) resistance mechanisms play a major role in temperature-modulated basal resistance to *Pst* DC3000 (Rev. #1 major issue 1).
2. In **new Fig. S4a-b** we quantify total SA by GC-MS and *PR1* expression in the five representative *A. thaliana* accessions in response to temperature to address concerns about SA variation measured by biosensor-based luminescence method (Rev. #2 major issue 1). These data reinforce our existing measurements (in Fig. 3a, Table 1 and Fig. S1) showing that the bioluminescence assay accurately reflects changes in SA amounts between accessions and temperatures.
3. In **new Fig. S4 c-e** we determine expression levels of key SA metabolic pathway genes *ICS1*, *PBS3* and *PAL4* in the same five representative *A. thaliana* accessions in response to temperature. The data show that changes in expression of these SA biosynthesis genes are not associated with the observed variation in anti-bacterial resistance between 16^o^C and 22^o^C (Rev. #1 major issue 3). We now discuss this in the context of reported more drastic high to moderate temperature differences which, in pathogen-triggered or autoimmune *A. thaliana* lines, involve *ICS1* and *PBS3* expression changes.
4. In **new Fig. S9**, we measure expression levels of *bHLH059* (prev. *UNE12*) in five *A. thaliana* accessions from different phenogroups in response to temperature. These data strengthen our claim that natural variation of *bHLH059* expression rather than protein sequence polymorphisms leads to phenotypic variation in temperature-dependent immunity patterns (Rev. #1 major issue 2).
5. In **new Fig. S10**, we show images of two independent *bHLH059* T-DNA insertion lines with mature siliques to address concerns about sterility raised by the original gene name „unfertilized embryo sac 12 (Rev. #2 major issue 7). We now refer to *UNE12* as *bHLH059* throughout the paper.

Below we answer all reviewer comments and highlight corresponding adds or changes in the ms text in yellow.

We hope that this revision will be considered suitable for publication in PLoS Genetics.

With best wishes, Jane E. Parker (senior corresponding)

PS. If accepted, please can we have Friederike Bruessow (first author) as co-corresponding author with me. She is submitting the revision material.

**Reviewer #1:**
 MAJOR ISSUES:
1) One key issue that the authors need to absolutely resolve is their exclusion of “stomatal resistance as contributing significantly to the temperature effects observed on bacterial infection in these 11 accessions” (Line 186-188). To properly delineate the stomatal vs. apoplastic contributions of the plant defense responses, the authors need to perform direct apoplastic infiltration of Pst DC3000 in Figures 3 and 6, and also show that the same trends prevail when compared to spray-inoculations. The 4h monitoring of Pst DC3000 levels after spraying (Figure S4) only represents a single timepoint and does not exclude the stomatal dynamics before or after that timepoint. Because the conclusion on post-stomatal resistance is at the crux of this paper, the infiltration experiments are crucial.

***Response:*** *Thanks for highlighting this. We take on board the reviewer’s point that we can’t rule out a contribution of stomatal entry to the observed natural variation in some of the 105 accessions. We modified the text accordingly and discuss (line 236). To address the possible role of stomatal resistance dynamics in altering bacterial growth in leaves of different accessions in response to temperature, we performed a bacterial apoplastic infiltration experiment with additional time points in five representative differential A. thaliana accessions at 16^o^C and 22^o^C. Data are shown in* ***new Fig S7****. Based on this and the existing data (Fig. S5a) and new Fig. S4, see below) we argue that apoplastic (post-stomatal) resistance mechanisms play a major role in temperature-modulated SA and basal resistance to Pst DC3000.We show that with infiltration and spray infection we have uniformly low bacteria loads in leaves across genotypes and between temperatures (see Fig S5a and S7), suggesting that circumventing stomatal resistance by infiltration in selected accessions at the different temperatures does not alter Pst DC3000 infection trends. Assessing multiple time points in the infiltration experiment allowed us to see that the final trends at 4 dpi are already discernable at 24 hpi in most of the tested lines (Fig. S7).*

2) Another key issue that the authors need to validate is the lack of amino acid mutations in the UNE12 gene between the various accession phenogroups. As stated in lines 297-299, “Since this SNP leads to a synonymous mutation, we reasoned that variation in expression of UNE12 rather than protein sequence might underlie temperature-modulated SA and/or bacterial resistance.” To validate this conclusion and because the GWAS identification of UNE12 is the main thesis of this paper, the authors need to show differences in UNE12 gene expression (by RT-qPCR) between the various phenogroups of Arabidopsis accessions. In relation to this, the authors could provide details if there are also polymorphisms that lie within the UNE12 gene promoter and show if putative regulatory control elements are affected by the nucleotide change/s (if applicable).

***Response:*** *We measured UNE12 (now referred to a bHLH059; Rev#2) expression in response to temperature in five key accessions: Ven-1 and Mz-0 (increased SA with increased temp), Fei-0, Ei-2 and Col-0 (decreased SA with increased temp) in* ***new Fig. S9****. (line 335). This shows that UNE12 expression patterns are different between phenogroups, strengthening our claim that natural variation of bHLH059 expression rather than protein sequence polymorphisms underlie phenotypic variation in temperature-dependent immunity patterns.*

*We show variation in the UNE12 promoter within 1 kb upstream of the gene in Fig. 5c but we cannot exclude other regulatory elements in cis or trans playing a role in expression. We investigated sequence conservation in UNE12 and its full promoter up to the neighbouring gene in related Brassicacea using VISTA to define important regulatory elements with MEME. This wasn’t conclusive and requires more analysis, so we don’t mention in the ms.*

*We did find a SNP that results in a CGATT intronic enhancer motif ^1^ in the first intron of UNE12 present in Fei-0, Ei-2 and most accessions belonging to this phenogroup that might explain higher UNE12 levels at 22°C (Fig. S9). It’s unclear why Col-0, without this motif, shows UNE12 expression of this phenogroup.* *The mechanism by which UNE12 expression and function is modulated in different accessions and the effects of temperature is an interesting aspect we would like to follow up in future work.*

1.	Rose, A. B., Carter, A., Korf, I. & Kojima, N. Intron sequences that stimulate gene expression in Arabidopsis. *Plant Mol. Biol.* **92**, 337–346 (2016).

3) Finally, to add additional mechanisms to the temperature-sensitive basal SA levels at 16°C vs. 22°C, the authors could demonstrate which metabolic pathway mediates this phenomenon. Mang et al. (2012 Plant Cell 24) showed that the temperature sensitivity of basal SA levels at 22°C vs. 28C is due to differences in basal ICS1 gene expression. Because the authors show that the difference between 16°C and 22°C is in the basal SA levels, it would be good to resolve if this is due to the ICS1/PBS3 pathway as well by measuring gene expression levels.

***Response:*** *Thanks for flagging this. We measured expression of ICS1, PBS3 and PAL4 (as a major gene in the alternative SA synthesis pathway) in response to temperature in five key differential accessions (Ven-1, Mz-0, Fei-0, Ei-2 and Col-0 ) shown in* ***new Fig. S4c-e****. In this experiment, PR1 expression shown in* ***Fig S4b*** *mirrors SA patterns measured by GC-MS in* ***Fig. S4a,*** *but ICS1, PBS3 or PAL4 do not show temperature-related expression changes in these tissues, suggesting that temperature-dependent SA accumulation is at another level, which is interesting. In the revised ms we discuss these data in the context of reported more drastic high to moderate temperature differences which, in pathogen-triggered or autoimmune A. thaliana lines, involve ICS1 and PBS3 expression changes (line 185).*

MINOR ISSUES:
Figure 5: Col-0 and Est-1 belong to one phenogroup (higher SA at 16°C than at 22°C), while Ven-1, Mz-0 and Nok-3 belong to another phenogroup (higher SA at 22°C than 22°C). However, they all do not have polymorphisms in UNE12. Can the authors provide possible explanations for this in the Discussion?

***Response:*** *This is correct. Although cis regulatory elements are most common, transcription is also affected by trans regulatory elements which are hard to define at this stage.*

*We discuss this along with new UNE12 expression data from* ***new Fig. S9*** *(line 335). Please also see response to major point 2.*

In Lines 439-441, the authors emphasize that Mz-0 has an hyperactive ACD6 allele. They could also mention that this is the case with Est-1, which belongs to another phenogroup.
***Response:*** *We have added this information (line 483)*

Specific comments on Figures and Tables:
• Figure 7: It is mentioned that “n=14 from three biological replicates” – was one experiment missing a sample? Please clarify. ***Response:*** *Typo n=12 (4 samples/replicate)*
• Suppl Figure S1: Was this one independent experiment? Or was this experiment repeated? ***Response:*** *Data for Fig. S1 comes from one independent experiment. As the scope of this experiment was to compare different SA measurement methods and not the variability of SA contents in different genotypes, we analyzed 5 replicates from the same experiment rather than from biological replicates. In the revised ms, data in* ***new Fig. S4*** *further reinforce our conclusion that the bioluminescence assay reflects changes in SA amounts between accessions and temperatures.*

• Suppl Figure S2: Which 15 accessions were used? ***Response:*** *We used all accessions that we further characterised in Figs 2 and 3. We have added info to the legend of Fig. S2.*
• Suppl Figure S6: What is the extra bar in Ven-1? ***Response:*** *We explained this in the legend - these are two independent heterozygous Ven-1/NahG lines* ***.*** *We use these because Ven-1 generation time is extremely long and we wanted to perform SA extraction under the same conditions as the other lines. As NahG enzymatically transforms SA to catechol, only one copy of the gene is sufficient as long as it is translated.*

• Suppl Figure S7b and c: There are larger variations in SA and PR1 levels in the une12 mutant compared to those in Col-0. Are there samples that are almost outliers in the une12 measurements? ***Response:*** *We think the large variation in une12 (bhlh059) KO lines might be due to compensatory effects of its paralogue At1g03040. We added a mention in the discussion (line 530). We are currently characterising the single and double mutants. They display developmental phenotypes in flowering time and inflorescence architecture but since we didn’t get consistent results in the double mutants so far, analysis will take much more time.*

• Suppl Table S4: Please indicate primer annealing temperatures used. ***Response:*** *Done*

Specific comments on the text:
• Line 2: A title suggestion could be “Natural variation in temperature-modulated immunity uncovers transcription factor UNE12 as a thermoresponsive regulator in Arabidopsis thaliana” ***Response:*** *Changed (line 1)*

• Line 18: Rephrase “… endogenous pathways remains unclear….” to “… endogenous pathways is not fully characterized….” There have been various studies (i.e. PhyB, PIFs, H2A.Z and NLRs) showing how temperature intersects with endogenous pathways. ***Response:*** *Adapted (line 25)*
• Line 22: In the line “…limiting bacterial pathogen,” please specify the pathogen. ***Response:*** *Changed (line 29)*
• Line 60: Please mention that temperature can intersect with the HSP90 proteins (Wang et al., 2015 Nat Commun 7), SIZ1 E3 ligase (Hammoudi et al., 2018 PLoS Genetics 14) and the DET1/COP1 photomorphogenic regulatory module (Gangappa and Kumar, 2018 Cell Rep 25) ***Response:*** *Thanks.* *We added findings of Hammoudi 2018 and Gangappa 2018 to paragraph on temperature-dependent growth-defense regulation. However, we don’t cite Wang et al., 2015 Nat Commun 7 because it highlights stabilisation of auxin signalling by HSP90 as a growth regulator at high temperatures without investigating its implication on defense, which ist the main focus of our paper. (line 69)*

• Line 70: Please mention that PTI can also be negative regulated by higher stress temperatures when measuring different defense outputs (Janda et al., 2019 Mol Plant Pathol 20). ***Response:*** *We added theses findings to the text (line 85)*

• Line 79: Fix the citation number “19” as a superscript. ***Response:*** *Changed (line 90)*
• Line 98: Change “… variation in immunity responses…” to “… variation in immunity and growth responses…” ***Response:*** *Changed (line 110)*

• Line 118: “Biomass” is both singular and plural. ***Response:*** *Changed throughout MS*
• Line 843: Change to “Manhattan plot”. ***Response:*** *Changed (line 1002)*

**Reviewer #2:**
**Major points:**
1. Huge variation is observed in the SA level measurements in Table S1, some with >10-fold differences among repeats. Therefore it is critical to corroborate the important SA levels data for the selected ecotypes (in Fig 2/3/4/6) using the more accurate HPLC-based or other methods. This will reduce the variations and hopefully error bars to reveal the trends better. The authors should also indicate in the legends how many times the quantitative experiments are repeated.

***Response:*** *We show in Fig. S1 that the biosensor-based method actually reduces variability between SA measurements compared to the established and accurate GC-MS method. Maybe because we have fewer extraction and processing steps with the biosensor-based method. We adapted the legend to clarify experimental repeats. We repeated quantification of total SA contents in five key differential accessions by GC-MS and show results in* ***new Fig. S4a****. (line 185). These additionnal assays show that Ven-1 and Mz-0 display higher SA contents at 22°C compared to 16°C while Fei-0, Ei-2 and Col-0 show the opposite trend, corroborating our initial findings (Fig. 3a and Table S1). We feel these data emphasize the reliability of the biosensor-based method.*

2. Corresponding to Fig 2c/d/e/f which were collected at 22C, the authors should show respective data at 16C to strengthen their conclusions.

***Response:*** *The particular accessions shown in Fig. 2c-f do not show the same growth x defense tradoff characteristics at 16°C as at 22°C. Additionnally the negative correlation between plant fresh weight and SA content is even lower at 16°C (R=-018 Fig. 2ab) and the plant sizes vary on a much smaller scale compared to plants grown at 22°C (Fig. 2ab). This made it impossible to select sufficient accessions with significantly diverse combination of traits to conduct a reciprocal analysis.*

3. A graph can be added to Fig 2 to summarize Table S1 and support the authors’ conclusions on SA-growth tradeoff, with X-axis being ecotype names and Y-axis being the ratio of SA to fresh weight (SA/FW). The curve at 16C can be arranged to be incremental, while the curve at 22 would be random.

***Response:*** *We summarized the data in Table S1 by showing the relationship between biomass and SA contents as a scatterplot in Fig. 2a-b for each temperature separately, representing the SA-growth trade-off by calculating Pearson’R. In both cases R is very low. We see a small negative correlation between SA and FW that is significant but not all ecotypes display a trade-off as we detail in Fig. 2c-f. After having tried different ways of representing the data, this seemed the most accurate and clear to us. We also tried to represent trade-off data as a SA/FW ratio, as you propose, but many lines end up having similar ratios depite displaying radically different phenotypes. See example below. We therefore prefer to stay with our representation in Fig. 2.*

| SA (ug/gFW) | FW(g) | SA/FW |
| --- | --- | --- |
| 4 | 2 | 2 |
| 1 | 0,5 | 2 |

4. In the results section titled genetic variation in A. thaliana SA-growth tradeoffs, it is stated that Ven-1, PHW-13 and Kas-2 had high total SA amounts and high biomass. However, according to the data shown in Table S1, total SA amounts of PHW-13 was quite low (<0.3 μg/g FW) at both temperatures. Please explain why it can be classified into high SA, high biomass group. Also, in Fig. 2d, the total SA amount of Ven-1, PHW-13 and Kas-2 were comparable, which is not consistent with the data on Table S1, please explain.

***Response:*** *Thank you for pointing this out. Going back to the original data, we realized that PHW-13 name and geographic origin had been inverted with Pa-2. Probably a human error in excel as these accessions follow each other alphabetically. We carefully checked. The corrected Table S1 is now consistent with data shown in Fig. 2 c-f*

5. In the results section titled genetic architecture of SA regulation by temperature, it is stated that 99 accessions were used to perform temperature x SA association analysis. Why not use all 105 accessions? Please explain.

***Response:*** *The GWAPP application was published after we started the temperature x SA screen. Not all accessions had been added to the App because of sequencing quality issues or other reasons, so six of our chosen accessions are missing from the App* ***parameters/tool***.

6. In Fig. 6 and Fig.7, a UNE12 T-DNA insertion line, une12-13, was used to perform the assays. But in the end of results section, another T-DNA insertion line, une12-01, was mentioned. It is stated that these two lines behaved similarly. So, what’s the reason here using another T-DNA line? Please clarify.

***Response:*** *In Fig. S10 we tested SA, defence and growth phenotypes of an independent bHLH059 T-DNA insertion line (now called bhlh059-01) to be sure that these are consistent and can be attributed to knockout of bHLH059.*

7. Although UNE12 is the name associated with the bHLH TF studied, it is quite misleading as the mutants do not seem to exhibit lethality or sterility. The inherited UNE conclusion is likely incorrect as it was from an early large scale screen. I would suggest the authors to use bHLH59 instead (see Heim et al 2003), and just mentioned that it also carries the UNE12 name (it might be worthwhile to show in a supplementary figure that there is no obvious sterility/lethal phenotype as suggested by UNE, just to clarify the literature).

***Response:*** *Thanks for pointing this out. We agree and have changed text, legends and figures accordingly****.*** *We also added pictures of bHlH059 mutants with mature siliques in* ***adapted Fig. S10f*** *to rule out any doubt about their fertility.*

Minor points:
The figures can be annotated better to assist with reading. For example, In Fig 2a/2b, the ecotypes selected for careful analysis should be indicated in the plots. The colours of Fig 2c/d/e should be explained (green/grey/purple) in the legend. ***Response:*** *Done*

Line 354 – Reference numbers should be superscript. ***Response:*** *Changed*

References section – The names of cited paper are not consistent. For instance, in line 586, all the initials are capitalized. But in line 590, only the initial of first word is capitalized.

***Response:*** *We inquired whether plos genetics needs this changed. Plos genetics keeps the format of the references as they come from providers of DOI. So we left things as they are.*
