# Supplementary material for: Natural variation in temperature-modulated immunity uncovers transcription factor bHLH059 as a thermoresponsive regulator in Arabidopsis thaliana
Source: PLoS Genet. 2021 Jan 25;17(1):e1009290. doi: 10.1371/journal.pgen.1009290 (PMC7861541; doi:10.1371/journal.pgen.1009290)
Supplement: Attachment — Submitted filename: Bruessow_Editor & Reviewer response R2.docx [file pgen.1009290.s016.docx]

**Response to Editor & Reviewers for** PGENETICS-D-19-02017R1

dear Dr. Mauricio

Thank you for passing on the reviewer comments to our revised ms. We provide a detailed response to all reviewers’ points below and mark changes in text in blue. We hope the ms is now suitable for publication.

Sincerely, Jane Parker (corresponding)

**Editor comment:** Both reviewers and the associate editor raise questions about the results presented in figure 6. Reviewer 1 suggests that a 2 way ANOVA would be the most appropriate first analysis rather than 2 separate 1 way ANOVA. You should do that analysis. Both reviewers raised issues about the clarity of that figure as well and you should do what you can to amke that figure as clear as possible.

*Thanks to reviewers for pointing this out. For better visual clarity in Fig. 6 data we have grouped genotype data by temperature and colour-coded genotypes, similar to the data display in Fig. 7. After performing a 2 way-ANOVA on expression of BHLH059, PIF4 and PR1, SA contents and PstDC3000 bacterial counts (0 and 4 d post infection) - influenced by genotype and temperature in the Fig.6 set of mutant lines, we determined that temperature significantly affects all our variables except for BHLH059 expression where it is marginally significant (p-value =0.08) and bacterial counts at 0 dpi, in line with results shown in Fig. S5. We decided it’s most informative and clear to examine differences between mutant lines within each temperature regime. Differences between genotypes within each temperature regime were obtained using Tukey’s multiple testing correction and are represented in new Fig.6. In Results we edited text: line 352 (marked in blue). Revisions to Fig. 6 legend are also marked in blue (line 1022). Our key conclusions remain the same - that mis-expression of bHLH059 in Col-0 disturbs temperature effects on SA and bacterial pathogen resistance, and that bHLH059 mutant and over-expression phenotypes are not related to PIF4/PIF5 effects*

2) Upload a Striking Image with a corresponding caption to accompany your manuscript if one is available (either a new image or an existing one from within your manuscript). If this image is judged to be suitable, it may be featured on our website. Images should ideally be high resolution, eye-catching, single panel square images. For examples, please browse our [archive](http://www.plosgenetics.org/article/browse/volume). If your image is from someone other than yourself, please ensure that the artist has read and agreed to the terms and conditions of the Creative Commons Attribution License. Note: we cannot publish copyrighted images.

- *done. Original image and caption.*

**Reviewers’ comments to the Authors:**

**Reviewer #1:**

To polish this manuscript, there are a few minor issues that the authors need to address as outlined below:

• If another revision is requested by the Editors, please make sure to include line numbers as was the case in the first version.

- *Sorry for the omission. Done.*

• Introduction, second paragraph (page 3): It would be great to update your literature review with the recently identified ELF thermosensing mechanism (Jung et al., 2020; <https://www.nature.com/articles/s41586-020-2644-7>).

*- We add a sentence in Introduction (ln: 74) to highlight this new paper, Ref. 14.*

• “High SA accumulation..” section (page 10): Please qualify the concluding sentence of the paragraph by adding “in the 16°C-22°C range.”

*- done.*

• “SA underlies differential…” section (page 11): In your concluding sentence, you state “These data suggest that … that the isochorismate SA biosynthesis pathway is sensitive to the temperature range used here.” I suggest that this second clause be deleted because you did not see gene expression differences in ICS1 and PBS3.

*- Fair point. It could be misleading.*

• Figure 6: I just noticed that one-way ANOVA was performed separately for each temperature. To facilitate easier comparisons between the two temperature within each genotype, I believe a two-way ANOVA with Tukey’s HSD may be more appropriate. In its current version, the statistical presentation (of the mean separations as letters) can be a bit confusing. For example, in panels c, d and e – the Col-0 values at 16°C vs. 22°C are statistically significant with the student’s t-test but have the same ANOVA letters, while the bhlh059 mutant and estradiol lines have different ANOVA letters (comparing the two temperatures) but are not statistically different.

- *- Thanks for pointing out. For better visual clarity in Fig. 6 data we have grouped genotype data by temperature and colour-coded genotypes, similar to the data display in Fig. 7. After performing a 2 way-ANOVA on expression of BHLH059, PIF4 and PR1, SA contents and PstDC3000 bacterial counts (0 and 4 d post infection) - influenced by genotype and temperature in the Fig.6 set of mutant lines, we determined that temperature significantly affects all our variables except for BHLH059 expression where it is marginally significant (p-value =0.08) and bacterial counts at 0 dpi, in line with results shown in Fig. S5. We decided it’s most informative and clear to examine differences between mutant lines within each temperature regime. Differences between genotypes within each temperature regime were obtained using Tukey’s multiple testing correction and are represented in new Fig.6. In Results we edited text: line 352 (marked in blue). Revisions to Fig. 6 legend are also marked in blue (line 1022). Our key conclusions remain the same - that mis-expression of bHLH059 in Col-0 disturbs temperature effects on SA and bacterial pathogen resistance, and that bHLH059 mutant and over-expression phenotypes are not related to PIF4/PIF5 effects.*

**Reviewer #2:** The authors addressed most of my concerns. One small suggestion:

For Fig 6E, please clarify the statistic analysis in the legend. I am a bit confused on the a/b/c categories, some were deemed different, but they seem quite similar to me. Please clarify.

- *For better visual clarity in Fig. 6 data we have grouped genotype data by temperature and colour-coded genotypes, similar to the data display in Fig. 7. After performing a 2 way-ANOVA on expression of BHLH059, PIF4 and PR1, SA contents and PstDC3000 bacterial counts (0 and 4 d post infection) - influenced by genotype and temperature in the Fig.6 set of mutant lines, we determined that temperature significantly affects all our variables except for BHLH059 expression where it is marginally significant (p-value =0.08) and bacterial counts at 0 dpi, in line with results shown in Fig. S5. We decided it’s most informative and clear to examine differences between mutant lines within each temperature regime. Differences between genotypes within each temperature regime were obtained using Tukey’s multiple testing correction and are represented in new Fig.6. In Results we edited text: line 352 (marked in blue). Revisions to Fig. 6 legend are also marked in blue (line 1022). Our key conclusions remain the same - that mis-expression of bHLH059 in Col-0 disturbs temperature effects on SA and bacterial pathogen resistance, and that bHLH059 mutant and over-expression phenotypes are not related to PIF4/PIF5 effects.*
